# Research on the mechanism of digital drive economy enhancing quality and efficiency

**DOI:** 10.1371/journal.pone.0316985

**Published:** 2025-02-05

**Authors:** Ruixvan Gao, Jialun Lyu

**Affiliations:** 1 Faculty of Foreign Languages, Beijing Language and Culture University, Beijing, China; 2 School of Economics, Beijing Technology and Business University, Beijing, China; Fooyin University, TAIWAN

## Abstract

This paper, based on unstructured data, examines the outcomes and impact mechanisms of digitalization from the perspective of technological advancement and institutional evolution, providing empirical evidence for the role of digitalization in promoting economic quality and efficiency. The study finds that digitalization contributes to the improvement of regional economic development efficiency. Its economic impact primarily manifests in the transformation of traditional value creation models and the enhancement of marketization levels. The digital economy, by activating digital platforms, alleviates the problem of information asymmetry, thereby enhancing the standardization of market transactions and improving economic efficiency.

## Introduction

**Fig 1:**
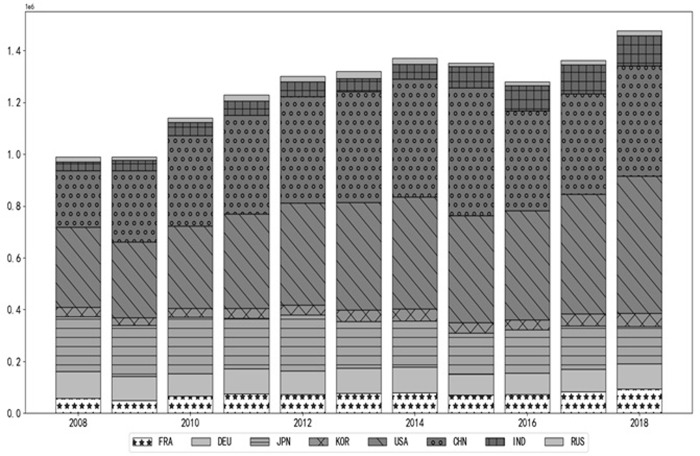
Value added of ICT industry in major countries (unit: Million US dollars).

The rapid advancement of digital technology and its increasingly significant influence are triggering a new wave of global technological revolution. As the digitalization process continues to progress worldwide, the impact of digital technology on the economic development of various countries is becoming more pronounced. For instance, developed countries like the United States, Japan, and South Korea have made remarkable achievements in digitalization, driving the transformation and upgrading of their economic structures. Meanwhile, emerging economies such as India and Russia are also actively promoting digitalization in hopes of achieving rapid economic growth and development. The development paradigm centered around the digital economy is playing an increasingly important role in representative industries worldwide. The ICT value added of major countries increased from $27,717.99 billion in 2008 to $38,627.38 billion in 2018, reflecting a growth rate of 39.36% (see Fig 1). China, in particular, has gained a significant first-mover advantage due to its recent emphasis on this sector. By 2018, China’s ICT value added ranked second globally, trailing only the United States.

In 2020, the State Council of China underscored the importance of data as a new factor of production in its "Opinions on Constructing a More Perfect Factor Market Allocation System," thereby formally recognizing the importance of ongoing informatization and digitalization in economic development. Every technological revolution profoundly affects the foundational organizational structures of production, embedding new technologies to create value and enhance efficiency. The first industrial revolution gave rise to factories, where mechanical production supplanted manual labor. The second industrial revolution led to the emergence of modern enterprises, which, through rational management division and departmental coordination, facilitated the effective utilization of internal and external resources to achieve economies of scale and scope.

Researchers currently explore whether digitalization can promote economic quality and efficiency from three main perspectives: First, it is believed that the development of digital technology can facilitate R&D collaboration among economic entities, optimize product development processes, and achieve cost reduction and efficiency improvement in economic development [1]. Second, some argue that digitalization has the effects of human capital investment and industrial structure upgrading, thereby strengthening total factor productivity [2,3]^.^ Third, digitalization is thought to enhance overall resource allocation efficiency by improving government governance capabilities [4]. Conversely, from the perspective of the Solow Paradox, some researchers contend that even high levels of digitalization do not necessarily enhance the performance of economic entities [5]. Additionally, constraints such as management experience, technological level, human capital, perceptions, and legal factors may render the digitalization process counterproductive, making the adoption of new technologies both an opportunity and a challenge [6]. Therefore, the impact of digitalization on economic efficiency is uncertain.

Moreover, existing research mainly focuses on the technical aspects, emphasizing how digitalization directly affects the economy through improved production efficiency, optimized economic management, and industrial upgrading. There is relatively limited discussion on how digital technology can improve the institutional environment and indirectly impact economic efficiency. The role of digitalization in enhancing economic efficiency is not only reflected in productivity improvement but also in potential adjustments to the institutional environment. Chinese society has typical characteristics of a relational society. As an informal institution, a relational society can mitigate the negative impacts of institutional deficiencies and information asymmetry by establishing relationships and mutual trust among economic entities. Digital technology provides new digital transaction platforms and a more transparent transaction environment for economic activities. Whether this helps transform the traditional relational transaction model at the institutional level, thereby further unleashing market vitality and improving economic efficiency, has not been well addressed in previous research.

In summary, although existing studies have recognized that the development and application of digital technology offer new possibilities for economic growth and productivity improvement, the role of digitalization in enhancing economic efficiency is complex and multi-dimensional. The ongoing debate centered on the Solow Paradox indicates that current research remains unclear and inconclusive regarding whether digital transformation can promote economic quality and efficiency. The technological progress perspective focusing on productivity improvement cannot fully explain the impact of digitalization on economic efficiency. There is a research gap regarding how digitalization can enhance economic quality and efficiency by influencing the institutional environment. Therefore, this paper constructs an analysis framework of "technological progress + institutional evolution" to explore the impact of digitalization on economic efficiency improvement.

### 1. The theoretical logic of the digital economy driving economic development and improving quality and efficiency

The development of the digital industry has enriched the variety of digital technologies and lowered the threshold for their use [7]. With the expansion of the reach and reduction in the cost of digital technologies, the potential of digital platforms has been activated. Digital technologies are increasingly embedded in the production organization and operations of various industries, transforming the way economies function. Digitalization refers to the process of integrating digital production factors into production, utilizing digital platforms to incorporate digital technologies into target industries. Enterprises restructure products and services, business processes, organizational structures, and business models through a combination of computing, communication, and connectivity technologies, thereby creating and capturing more value [8].

From a technological perspective, digital technology directly drives the transformation of business value creation models through the deep integration of innovative platforms with existing production resources. Its promotion of productivity enhancement is mainly reflected in the following:the high plasticity of digital technology facilitates lean production by reducing waste and inefficiency in production. According to lean production theory, the core goal of lean production is to identify and eliminate various wastes (Muda) [9,10]^.^ Economic entities can develop highly customized functions based on digital platforms, seamlessly integrating them into production resources and processes [11]. This functional extension endows production resources with "digital materiality"[12], which means that through the integration of digital capabilities, traditional production resources can not only accomplish physical production tasks but also achieve more efficient feedback through data feedback, optimization, and automated operation, thereby laying the foundation for lean production. For example, in Germany’s Industry 4.0, intelligent production equipment achieves real-time monitoring and adaptive adjustment of the production process through digital technology, significantly improving production efficiency by reducing redundant processes and rationally arranging work schedules.

From the perspective of institutional evolution, digitalization has profound impacts on economic and social development, primarily by alleviating information asymmetry problems. Digitalization provides solutions to information asymmetry issues, facilitating a shift in economic governance from relational to rule-based models. For instance, e-commerce platforms like Alibaba and Amazon reduce information asymmetry between buyers and sellers by providing detailed product information, user reviews, and seller credit ratings. These platforms’ search and recommendation systems also help users quickly find the needed products, increasing transaction efficiency and making platform transactions mainstream. Relational governance emphasizes personal connections between social individuals, significantly influencing corporate behavior and supply chain relationships [13]. However, as the scale of transactions and the overall economic volume increase, relational transaction models may increase economic operation risks, constrain the choice space of economic entities, and weaken their bargaining power. Rule-based, market-oriented transaction models gradually become the optimal choice for efficient economic development [14].

Digitalization weakens the relational governance mode through multiple channels, enhances the rule-based governance, and reduces the difficulty of governance mode transformation. Firstly, digital trading platforms enhance the extendibility of soft information, helping to gradually eliminate asymmetric information problems [15]. Digitalization leaves traces of circulating information in the network, transforming them into data. Digital trading platforms can utilize their advantages to collect information, establish relevant measurement systems, and process soft information into hard information. Secondly, digitalization reduces the governance costs of issues derived from information asymmetry. Digital platforms enrich economic governance methods and governance subjects, reducing the impact of asymmetric information problems. In addition to making judgments themselves, platform operators can also introduce other platform users for supervision. Platform sanctions will significantly increase the transaction costs of the sanctioned party, thereby restraining the behavior of market participants. Furthermore, digital platforms have the characteristic of "light assets," reducing the necessity of investing in physical assets, lowering market governance costs, and enhancing the level of economic standardization.

So, through the dual pathways of technological innovation and institutional reform, digitalization promotes the quality and efficiency of the economy, injecting new impetus into economic development. It not only directly optimizes the production process, but also promotes the optimization of economic structure and the improvement of efficiency by improving information flow and market governance mechanisms.

The neoclassical theory introduces exogenous technological factors as an important source of economic growth, while endogenous economic growth theory further regards technological progress as an endogenous process based on neoclassical theory, suggesting that continuous technological advancement will lead to sustained improvement in economic performance. This explains why digitization drives economic quality and efficiency improvement from a technological perspective. However, from an impact perspective, the digitization process can promote economic quality and efficiency improvement by breaking down relational transactions and establishing and improving rule-based transactions, a mechanism not covered in traditional economic theories. To provide a more precise description of the relationship between digitization and economic quality and efficiency improvement, this paper constructs a mathematical model to theoretically describe this issue.

The problem of information asymmetry allows the supply and sales side that connects with the company to obtain additional profits through speculative actions in the process of trading with the company. To avoid the loss of benefits caused by speculative behavior on the supply and sales side, companies will choose to maintain trading relationships with a small number of "acquaintances" to control potential speculative risks.

The total profit that a company can obtain is:

$$p_{total}=effort+\varepsilon$$ (4.2.1)

In this context, the *effort* refers to the supplier’s willingness to pay, which is inversely proportional to opportunistic behavior. The external factor *ε*, which affects profit fluctuations, is assumed to follow a normal distribution with a mean of 0 and a variance of σ^2^ for simplification of the analysis, σ^2^ reflects the degree of uncertainty in the business operations of the enterprise. The net profit *p*_*net*_ that the enterprise can obtain is represented by:

$$p_{net}=p_{total}-c\left(d,p_{total}\right)$$ (4.2.2)

Enterprises want to maximize the expected value of their net profit:

$$E\left(p_{net}\right)=effort-E\left(c\left(d,p_{total}\right)\right)$$ (4.2.3)

Where *c*(*d*, *p*_*total*_) represents the total cost paid to suppliers, the cost level is determined by the degree of digitalization *d* and the total profit *p*_*total*_ of the enterprise. The value of *d* ranges from [0,1], satisfying $c_{d}^{\prime }\left(d,p_{total\;}\right)<0,c_{p_{total}}^{\prime }\left(d,p_{total}\right)>0$. The cost can be divided into two parts:

$$c\left(d,p_{total}\right)=a+f\left(d,p_{total}\right)$$ (4.2.4)

Where *a* is the base market cost, Actually, the base market cost should also be a function of total profit. However, for the sake of simplifying the analysis, it is assumed to be a constant. This assumption does not affect the final conclusion, *f*(*d*, *p*_*total*_) represents the additional enterprise losses caused by supplier opportunistic behavior. This loss is determined by two factors: the degree of digitalization of the enterprise and the scale of transactions conducted by the enterprise. The larger the transaction scale for profit, the higher the losses due to supplier opportunism. However, increased information transparency can effectively mitigate supplier opportunistic behavior.

The indirect utility function facing suppliers satisfies an exponential form structure:

$$U=-e^{-R*c}$$ (4.2.5)

The supplier is risk averse, facing wealth acquisition risk that follows a normal distribution N(0,1). The supplier wants to maximize the expected value of its utility:

$$E(U)=E\left(c\left(d,p_{total}\right)\right)-R/2Var\left(c\left(d,p_{total}\right)\right)-fatigue(effort)$$ (4.2.6)

Among them, R>0 is the risk aversion coefficient of the supplier, which represents the utility loss brought by the supplier’s due diligence work and is a convex function.

Without loss of generality, suppose the additional loss of the enterprise is a linear function:

$$c\left(d,p_{total}\right)=a+(1-d)*b*p_{total}$$ (4.2.7)

Where b > 0 is the incentive coefficient for supplier opportunism. Substituting Eqs 4.2.7 into 4.2.6, the expected utility of the supplier can be expressed as follows:

$$E\left(a+(1-d)bp_{total}\right)-R/2Var\left(a+(1-d)bp_{total}\right)-fatigue(effort)$$ (4.2.8)

According to the properties of variance and expectation calculation, the above formula can be simplified to:

$$a+(1-d)b*effort+R/2b^{2}{\left(1-d\right)}^{2}\sigma^{2}-fatigue(effort)$$ (4.2.9)

In formula 4.2.9, the size of *b* depends on the degree of market completeness, with the incentive for supplier speculative profits being inversely related to the degree of market completeness. Enterprises usually have two ways to control the risk of trading speculative behavior: first, according to the incentive compatibility principle, they sign contracts with trading partners to constrain behavior through speculative compensation; second, by trading with "acquaintances" and mastering the speculative level of "acquaintances" in repeated games, making trading risks controllable. Under the condition of market completeness, that is, when speculative behavior no longer exists, enterprises only need to pay the market cost *a*. To ensure the reduction of speculative behavior caused by information asymmetry among suppliers, the conditions required are:

$$fatigue^{\prime }(effort)=(1-d)b$$ (4.2.10)

Higher supplier speculation incentives result in higher costs for suppliers to perform their functions normally. However, the degree of digitization can offset the impact of supplier speculation incentives. The higher the degree of digitization, the greater the weakening of supplier speculation incentives, and the more effectively it can mitigate the consequences of supplier speculation.

In the entire transaction, the enterprise’s residual can be expressed as:

effort(1−(1−d)b)−a (4.2.11)

Considering the supplier’s utility function, the basis for the establishment of the trading relationship is:

$$a=fatigue(effort)+\left(Rb^{2}\sigma^{2}\right)/2-b*effort$$ (4.2.12)

By combining Eqs 4.2.10–4.2.12, the residual of the enterprise can be obtained as:

$$effort-fatigue(effort)-R\sigma^{2}{\left(fatigue^{\prime }(effort)\right)}^{2}/2$$ (4.2.13)

Under the balanced condition, suppliers’ willingness to pay must satisfy the basic first-order condition.


$$fatigue^{\prime }\left(effort^{*}\right)=\frac{1}{1+A\;fatigue^{\prime \prime }\left(effort^{*}\right)\sigma^{2}}$$ (4.2.14)

As known from 4.2.10: $fatigue^{\prime }\left(effort^{*}\right)=\left(1-d^{*}\right)b^{*}$.

For a company in operation, under conditions of incomplete information, the value of b^*^ is unknown. It is only through repeated interactions that the company can obtain an observed value of b^*^. Therefore, companies are reluctant to expand their trading partners, which limits the expansion of transaction scale and is detrimental to enhancing their competitiveness. However, when the degree of digitalization is relatively high, companies can more quickly obtain information about their trading partners and determine the specific level of b^*^ Additionally, digitalization can weaken the incentive for supplier opportunism, thereby reducing the impact of b^*^.

This model considers the speculative behavior of distributors as a spontaneous action for stabilizing their own income. The profit loss of the enterprise originates from the uncertainty of the transaction volume with distributors during the trading process. The solution to the problem is to enhance information transparency, so that the speculative behavior of distributors can be regulated or the trading relationship between both parties can be made more stable. This can directly reduce the losses of the enterprise in market transactions. Moreover, in a relatively transparent information environment, the enterprise can reduce the risk of distributors and establish trading relationships with distributors whose matching transaction costs approach market costs, thus helping to dissolve relational transactions.

Digitalization has increased the channels for companies’ information disclosure and their ability to access market information, allowing companies to more effectively match with upstream suppliers. Furthermore, digital trading platforms have accelerated the speed of information dissemination, alleviating the friction costs caused by information asymmetry and ultimately strengthening companies’ operational capabilities.

### 2. Empirical analysis

#### (1) Data source and processing

Digitization can affect the mode of economic development through both production channels and non-production channels. This article sets up the following model to explore this issue:

Ineffciency=βDigitial+γX+μ+α+t+ε

1. The dependent variable: Economic growth inefficiency. This paper calculates the inefficiency of urban economic growth using a random frontier model. The following model form is set as a measure of inefficiency in economic development:
$$Y=L^{\beta_{1}}K^{\beta_{2}}I^{\beta_{3}}F^{\beta_{4}}e^{v-u}$$
  Among them, *Y* represents the level of economic development, *L* represents the quantity of the labor force, and K represents the stock of capital. This article also draws on the practices of relevant scholars [16], considering the impact of urban infrastructure investment (*I*) and urban openness to the outside world (*F*). *v* represents the random error term and *u* represents the economic inefficiency term. Specifically, the level of economic development *Y* is represented by the real GDP of the city; the quantity of labor force is indicated by the number of employed people in the city; the stock of capital is calculated using the perpetual inventory method, with the formula from Goldsmith [17]: $K_{i,t}=K_{i,t-1}(1-\delta )+I_{i,t}/P_{i,t}$, where *K*_*i*,*t*_ represents the capital stock of city *i* in period *t*, δ represents the rate of capital depreciation, following the practice of relevant scholars with a value of 10.96% [18], *I*_*i*,*t*_ represents the amount of new fixed asset investment, and *P*_*i*,*t*_ represents the fixed asset price index; the infrastructure investment is represented by the city’s freight volume; and the openness to the outside world is represented by the actual utilization of foreign capital.
2. Core explanatory variables: Level of digitization. The level of digitization in a region directly affects the development of companies and is reflected in their annual reports. Referring to existing research [19], a specific method was used to extract information from the annual reports of listed companies to reflect the level of digital development. Initially, the Chinese vocabulary was used to split the sentences from the digital economy-related reports, State Council bulletins, and policy documents for 2017 and 2018, with the creation of a list of specialized nouns related to digital technology. Furthermore, a web crawler was used to collect public annual reports of A-share listed companies from 2012 to 2022, and the frequency of appearance of vocabulary from the list of specialized nouns in the annual reports of listed companies from 2012 to 2022was further counted, with this frequency serving as the standard for measuring digitization.
3. Control Variables: This article controls the proportion of university students in the city’s population to control human capital; controls the number of street lamps per ten thousand square meters and the number of hospital beds to control the level of municipal services; controls the length of city roads to control the level of urban commuting; controls exports and FDI of the province where the city is located to control foreign trade.

#### (2) Data analysis results

As shown in the Table 1, (1) The first column displays the results of a Mixed Ordinary Least Squares (OLS) analysis. (2) The second column presents the regression results based on a Random Effects Model, which accounts for random variations in the data. (3) The third column provides the outcomes of a Two-Way Fixed Effects Model, a method that controls for fixed differences across cities and over time. (4) The fourth column further incorporates a time trend component into the Two-Way Fixed Effects Model to capture trends that evolve over time.

**Table 1:** Benchmark regression analysis results.

	(1)	(2)	(3)	(4)
Digital	-19.683***	-23.519***	-23.219***	-23.519***
	(4.088)	(4.522)	(4.524)	(4.522)
Control Variables	Yes	Yes	Yes	Yes
Time trend	No	No	Yes	Yes
Time fixed effects	No	Yes	No	Yes
City fixed effects	No	Yes	Yes	Yes
R-squared	0.300	0.321	0.309	0.321

The symbols *** represent significance at the 0.01 level, ** represent significance at the 0.05 level, and * represent significance at the 0.1 level, respectively.

Across all analyses, this study finds that the coefficient of the impact of digitalization on inefficiency in economic development is negative and statistically significant at the 0.01 level. This indicates that digitalization can significantly reduce inefficiencies in economic activities, thereby supporting our research hypothesis: digital transformation enhances the quality and efficiency of the economy.

#### (3) Robustness testing

First, to address potential causal confusion between economic development and digitalization levels, we introduced the lagged digitalization level as a variable in our regression analysis. This approach utilizes data from the preceding period to mitigate the effects of simultaneous causal relationships. As shown in column (1) of Table 2, the results indicate that the enhancement of digitalization levels remains significantly associated with economic quality and efficiency improvements.

**Table 2:** Robustness test results.

	(1)	(2)	(3)	(4)	(5)
	Ineffciency	Ineffciency	Digital	Ineffciency	Digital
IV			2.484***		-30.093***
			(0.265)		(4.884)
Digital	-28.416***	-12.452***		-46.074**	
	(6.465)	(5.411)		(20.029)	
IMR	-	-	-	-	Yes
Control variables	Yes	Yes	Yes	Yes	Yes
Time trend	Yes	Yes	Yes	Yes	Yes
Time fixed effects	Yes	Yes	Yes	Yes	Yes
City fixed effects	Yes	Yes	Yes	Yes	Yes
			F_test	42.10***	
			Anderson LM	83.809***	
			C-D Wald F	87.232***	

Second, recognizing that provincial capital cities possess unique developmental conditions that could affect the consistency of coefficient estimates, we excluded these cities from our sample for analysis. This measure ensures that our conclusions are not biased by the specific developmental conditions of certain cities. The results, as depicted in column (2) of Table 2, continue to support our primary findings.

Third, to address potential endogeneity issues, we employed an instrumental variable, constructed by the interaction of whether a region implemented the Broadband China policy and the number of post offices per million people in 1984. The regional variability of the Broadband China policy provides an exogenous shock to the digitalization process, while the 1984 post office count reflects historical differences in information and communication infrastructure. As indicated in column (3) of Table 2, the direction of the regression coefficient remains unchanged and is statistically significant at the 0.01 level. This suggests that even when considering the instrumental variable, the positive effects of digital transformation remain robust.

Fourth, to address potential selection bias, we applied the Heckman two-step method. Initially, we used a Probit model to estimate the probability of digital transformation and then incorporated the inverse Mills ratio into the main regression equation to adjust for possible biases. As shown in column (5) of Table 2, after bias adjustment, the results remain robust.

Through these robustness checks, our findings demonstrate that digital transformation significantly promotes the enhancement of economic quality and efficiency under various conditions. This further strengthens the reliability and persuasiveness of our research conclusions.

#### (4) Mechanism verification

Digitalization reduces the incentive level for suppliers to speculate, thereby alleviating the constraints of relational transactions on corporate development. This article sets up the following model to study the digitalization impact mechanism:

relationship=digital*β+X*γ+t+τ+μ+ε

effciency=relationship*δ+digital*β+X*γ+t+τ+μ+ε

Asymmetric information leading to relational transactions is a significant factor causing efficiency losses. Therefore, on the one hand, this paper explores the direct impact of digitalization on business efficiency, and on the other hand, this paper investigates whether the process of digitalization improves efficiency by reducing information asymmetry and dismantling relational transactions. Where *relationship* represents the level of relational transactions in the market. This article reflects the level of relational transactions from the perspective of enterprises through customer concentration and supplier concentration, using the Supply Chain Specificity Index as a comprehensive reflection of the level of relational transactions. The rationale for this approach lies in the following points: Firstly, in relational transactions, information asymmetry is usually high because the relationships between enterprises are built on trust and long-term cooperation, making it difficult for outsiders to accurately assess. This aligns with a highly concentrated supply chain, as information is often controlled and shared by a few enterprises. Secondly, high concentration usually implies that enterprises rely on a few key partners within their supply chain. Such reliance is often based on long-term trust and personal relationships rather than mere market transactions. This effectively reflects the relational factors that enterprises rely on in their transactions. Lastly, relational transactions are typically accompanied by higher transaction costs, including the costs of negotiation, communication, and maintaining the relationship. In a highly concentrated supply chain, enterprises need to spend more resources managing and maintaining a few key partnerships, which also corresponds to the high transaction costs characteristic of relational transactions. *Digital* represents the level of digitalization of the company, which is characterized by the frequency of digital technology terms in the company’s annual report. *Efficiency* represents the operational performance of the company, which is characterized by the return on total assets. *X* Represents control variables.

As shown in Table 3, the results of the first phase indicate that digitalization significantly reduces the level of relational transactions. This implies that as the degree of digitalization within firms increases, their supply chains become more diversified and decentralized. In the analysis of the second phase, the enhancement of firm performance due to digitalization is equally significant. This supports our research objective: digital transformation not only directly improves operational efficiency but also further strengthens a firm’s competitive capacity by reducing information asymmetry. Moreover, the analysis results demonstrate that the decreased level of relational transactions contributes to the enhancement of firm performance, suggesting that a diversified supply chain structure has a positive impact on corporate development.

**Table 3:** Mechanism analysis results.

	(1)	(2)	(3)	(4)	(5)	(6)
	customer	JROA	supplier	JROA	supplychain	JROA
digital	-0.009*	0.002**	-0.003**	0.002**	-0.006**	0.002**
	(0.005)	(0.0009)	(0.001)	(0.0008)	(0.003)	(0.0008)
customer		-0.004***				
		(0.001)				
supplier				-0.020***		
				(0.005)		
supply chain						-0.010***
						(0.002)
Control Variables	Yes	Yes	Yes	Yes	Yes	Yes
Time Trend	Yes	Yes	Yes	Yes	Yes	Yes
Time Fixed Effects	Yes	Yes	Yes	Yes	Yes	Yes
Individual Fixed Effects	Yes	Yes	Yes	Yes	Yes	Yes

### 3. Conclusion and recommendations

This study investigates the impact mechanisms of the digital economy on traditional value creation models in the context of rapid digital transformation. Digitalization profoundly influences mainstream economic paradigms by establishing decentralized, integrated, automated, and low-consumption systems, thereby innovating the processes of value creation and capture, ultimately reshaping the economic landscape. From the perspective of technological progress, exploring the malleability of digital technologies and integrating them with traditional technologies can effectively improve production processes and subsequently enhance productivity.

From the perspective of institutional evolution, digital trading platforms reduce the level of relational transactions and increase the level of market transactions, thereby enhancing market standardization. Simultaneously, digitalization improves corporate profitability; companies with higher levels of digitalization exhibit lower concentration in their supply chains and distribution networks, which contributes to higher operational efficiency. The theoretical contributions of this study are as follows: (1) It extends the understanding of the relationship between the digital economy and economic efficiency. Through systematic analysis, the study reveals that digitalization not only directly enhances productivity through technological progress but also boosts overall economic efficiency through network effects that drive regional economic synergy. (2) It emphasizes the reshaping of market transaction methods and corporate profit models by digitalization, providing new perspectives for future research. (3) It explores the necessity of institutional evolution in the digitalization process, particularly the changes in market standardization and corporate organizational forms, enriching the theoretical framework of the digital economy.

Based on the above conclusions, the following recommendations are proposed:

The development of digitization is a comprehensive and multi-level process. Digitization should be pursued around the trinity of digital networks, digital innovation, and digital platforms. With digital innovation as the driving force, digital networks as the foundation, and digital platforms as the intermediary, digitization can be advanced in a reasonable and orderly manner.

1. Enhance Digital Construction and Improve Digital Infrastructure
  Improving digital networks begins with the enhancement of digital infrastructure. Digital infrastructure comprises a system of facilities based on communication networks and computing power. Scientifically and reasonably constructing a supportive digital network infrastructure is crucial. First, the construction should align with specific industrial and livelihood conditions while considering fairness and addressing the digital infrastructure needs of remote areas to narrow potential regional digital divides, such as providing special funds to support the construction of digital infrastructure in remote areas. Second, clearly define the production requirements for digital infrastructure-related equipment, striving for quality and quantity assurance. Third, formulate reasonable regional digital development strategies and establish a comprehensive digital public service system to promote interconnected and shared data and information through a digital public service system.
2. Utilize Digital Platforms as Intermediaries to Promote Innovation-Driven Development
  Digital innovation is the core driving force of the digital technology revolution and the backbone of continuous digital advancement. In the digital economy era, the innovation paradigm is also changing. With the application of digital technologies, innovation organizations and related innovation resources exhibit a trend of decentralization under the influence of digital innovation platforms. Data plays an increasingly important role in the innovation process. Therefore, it is essential to leverage the innovation-leading role of digital innovation platforms by fostering a favorable platform ecosystem, promoting the co-construction and sharing of digital innovation resources, and clearly defining the direction of digital technology development. This should be combined with the improvement of policy environments, the establishment of digital pilot projects, and the formation of digital technology innovation bases. Alternatively, establishing special funds or loan programs can support traditional enterprises in the technological upgrades and process improvements necessary for digital transformation.
3. Enhance Digitalization Support and Emphasize the Service-Oriented Development of Manufacturing Enterprises
  Encourage enterprises to implement concurrent service-oriented transformations. Service-oriented transformation is both a significant direction for modern enterprise development and a requirement for successful digital transformation. The industrial value of digitalization must be realized through providing solutions to consumers. Digitalization endows manufacturing enterprises with digital attributes for their products, offering value creation through software and hardware transformations. However, this transformation process often requires corresponding organizational resources and processes to further achieve value realization. The combination of service orientation and digitalization can provide optimal configurations for creating consumer solutions in complex economic environments. For instance, policies supporting the digitalization of small and medium-sized enterprises, providing training and technical support to help them adapt to new digital platforms, could be formulated.

## Supporting information

S1 Data (XLSX)

S1 Table (DOCX)
