# Supplementary material for: Research on the mechanism of digital drive economy enhancing quality and efficiency
Source: PLoS One. 2025 Feb 5;20(2):e0316985. doi: 10.1371/journal.pone.0316985 (PMC11798452; doi:10.1371/journal.pone.0316985)
Supplement: S1 Table — (DOCX) [file pone.0316985.s002.docx]

**Table 1. Benchmark Regression Analysis Results.**

|  | (1) | (2) | (3) | (4) |
| --- | --- | --- | --- | --- |
| Digital | -19.683*** | -23.519*** | -23.219*** | -23.519*** |
|  | (4.088) | (4.522) | (4.524) | (4.522) |
| Control Variables | Yes | Yes | Yes | Yes |
| Time trend | No | No | Yes | Yes |
| Time fixed effects | No | Yes | No | Yes |
| City fixed effects | No | Yes | Yes | Yes |
| R-squared | 0.300 | 0.321 | 0.309 | 0.321 |

^The symbols *** represent significance at the 0.01 level, ** represent significance at the 0.05 level, and * represent significance at the 0.1 level, respectively.^

**Table 2 Robustness Test Results**

|  | (1) | (2) | (3) | (4) | (5) |
| --- | --- | --- | --- | --- | --- |
|  | Ineffciency | Ineffciency | Digital | Ineffciency | Digital |
| IV |  |  | 2.484*** |  | -30.093*** |
|  |  |  | (0.265) |  | (4.884) |
| Digital | -28.416*** | -12.452*** |  | -46.074** |  |
|  | (6.465) | (5.411) |  | (20.029) |  |
| IMR | - | - | - | - | Yes |
| Control variables | Yes | Yes | Yes | Yes | Yes |
| Time trend | Yes | Yes | Yes | Yes | Yes |
| Time fixed effects | Yes | Yes | Yes | Yes | Yes |
| City fixed effects | Yes | Yes | Yes | Yes | Yes |
|  |  |  | F_test | 42.10*** |  |
|  |  |  | Anderson LM | 83.809*** |  |
|  |  |  | C-D Wald F | 87.232*** |  |

**Table 3 Network Effects of Digitalization**

|  | (1) | (2) | (3) | (4) | (5) | (6) |
| --- | --- | --- | --- | --- | --- | --- |
|  | Inefficiency | Inefficiency | Inefficiency | Inefficiency | Inefficiency | Inefficiency |
| Digital | -25.700*** | -6.086 | -37.394*** | -14.298*** | -23.081* | -2.900 |
|  | (4.197) | (5.384) | (11.930) | (4.079) | (13.585) | (5.152) |
| w_Digital | -11.136*** |  | -7.501*** | -8.590*** |  |  |
|  | (0.675) |  | (1.235) | (0.995) |  |  |
| Digital*w_Digital |  | -0.011*** |  |  | -0.021*** | -0.004** |
|  |  | (0.002) |  |  | (0.006) | (0.002) |
| Control variables | Yes | Yes | Yes | Yes | Yes | Yes |
| Time trend | Yes | Yes | Yes | Yes | Yes | Yes |
| Time fixed effects | Yes | Yes | Yes | Yes | Yes | Yes |
| City fixed effects | Yes | Yes | Yes | Yes | Yes | Yes |
| R_se | 0.416 | 0.334 | 0.419 | 0.546 | 0.405 | 0.499 |

**Table 4 Mechanism analysis results**

|  | (1) | (2) | (3) | (4) | (5) | (6) |
| --- | --- | --- | --- | --- | --- | --- |
|  | customer | JROA | supplier | JROA | supplychain | JROA |
| digital | -0.009* | 0.002** | -0.003** | 0.002** | -0.006** | 0.002** |
|  | （0.005） | （0.0009） | （0.001） | （0.0008） | （0.003） | （0.0008） |
| customer |  | -0.004*** |  |  |  |  |
|  |  | （0.001） |  |  |  |  |
| supplier |  |  |  | -0.020*** |  |  |
|  |  |  |  | （0.005） |  |  |
| supply chain |  |  |  |  |  | -0.010*** |
|  |  |  |  |  |  | （0.002） |
| Control Variables | Yes | Yes | Yes | Yes | Yes | Yes |
| Time Trend | Yes | Yes | Yes | Yes | Yes | Yes |
| Time Fixed Effects | Yes | Yes | Yes | Yes | Yes | Yes |
| Individual Fixed Effects | Yes | Yes | Yes | Yes | Yes | Yes |
